# Contribution of Endothelial Laminin-Binding Integrins to Cellular Processes Associated with Angiogenesis

**DOI:** 10.3390/cells11050816

**Published:** 2022-02-26

**Authors:** Hao Xu, Susan E. LaFlamme

**Affiliations:** Department of Regenerative and Cancer Cell Biology, Albany Medical College, 47 New Scotland Ave., Albany, NY 12208, USA; haoxu1@upenn.edu

**Keywords:** integrins, laminin, angiogenesis, gene expression

## Abstract

Endothelial cells engage extracellular matrix and basement membrane components through integrin-mediated adhesion to promote angiogenesis. Angiogenesis involves the sprouting of endothelial cells from pre-existing vessels, their migration into surrounding tissue, the upregulation of angiogenesis-associated genes, and the formation of new endothelial tubes. To determine whether the endothelial laminin-binding integrins, α6β4, and α3β1 contribute to these processes, we employed RNAi technology in organotypic angiogenesis assays, as well in migration assays, in vitro. The endothelial depletion of either α6β4 or α3β1 inhibited endothelial sprouting, indicating that these integrins have non-redundant roles in this process. Interestingly, these phenotypes were accompanied by overlapping and distinct changes in the expression of angiogenesis-associated genes. Lastly, depletion of α6β4, but not α3β1, inhibited migration. Taken together, these results suggest that laminin-binding integrins regulate processes associated with angiogenesis by distinct and overlapping mechanisms.

## 1. Introduction

Angiogenesis contributes to both normal and pathological processes, including tissue repair, cancer progression, and inflammation [1,2]. Angiogenesis is a multistep process that involves the sprouting of endothelial cells from the pre-existing vasculature, which then form endothelial tubes that anastomose with one another to form new vascular networks. Although many mechanisms and regulatory pathways have been identified, a further understanding of the underlying mechanisms that regulate specific aspects of new vessel formation remains an important objective.

At the onset of angiogenesis, endothelial cells interact with proteins present in the extracellular matrix, some of which are provided by other cell types, such as those present in the provisional matrix during tissue repair [3,4]. Endothelial cells themselves also secrete matrix proteins including fibronectin and the basement membrane components, laminin-411 and laminin-511. The interaction of endothelial cells with these adhesion proteins using members of the integrin family of adhesion receptors contributes to the formation and stabilization of endothelial tubes [3,5,6,7,8,9].

Endothelial cells express several integrin heterodimers, including the three laminin-binding receptors α3β1, α6β1, and α6β4, which are known to bind laminin-511 [3,9,10,11]. Global deletion of the α3 subunit (Itga3), the α6 subunit (Itga6), or the β4 subunit gene (Itgb4) in mice demonstrated that integrins α3β1, α6β1, and α6β4 are not required for developmental angiogenesis [12,13,14,15,16,17]. To study the role of these integrins during angiogenesis in the adult, several labs have used the conditional endothelial deletion of either the integrin α3 subunit, the α6 subunit, or the β4 integrin subunit gene. The loss of expression of the α3β1 integrin has led to enhanced pathological angiogenesis, suggesting that endothelial α3β1 functions to inhibit angiogenesis [15]. The loss of α6 integrins has been reported to either promote or inhibit angiogenesis, depending upon whether α6 alleles were targeted by the expression of either a Tie1- or Tie2-driven Cre recombinase [16,17,18,19]. The mechanisms responsible for these disparate phenotypes are not fully understood; however, the phenotype of mice expressing the Tie2-driven Cre recombinase exhibited defects due to the loss of the expression of α6 integrins not only in endothelial cells, but also in macrophage and endothelial progenitors [16,17,18,19]. It has been difficult to distinguish contributions from α6β1 and α6β4 during angiogenesis, as the integrin β1 subunit dimerizes with multiple α subunits in endothelial cells, making it difficult to discern functions specific to α6β1 when α6β4 is also expressed. Mouse genetic studies examining the effect of Tie2-dependent deletion of the β4 subunit have not focused on angiogenesis per se; however, these studies have identified a role for α6β4 in hypoxia-induced vessel remodeling and in promoting endothelial barrier function in the brain vasculature in response to inflammation [20,21]. Others have demonstrated that α6β4 is expressed by angiogenic vessels and that the global deletion of the signaling portion of the β4 subunit cytoplasmic domain inhibited tumor angiogenesis [22]. Nonetheless, the contribution of endothelial α6β4 to angiogenesis has not been directly examined in the adult, and is complicated by the disparate reports of the restricted expression of the α6β4 integrin to a subset of endothelial cells [20,22,23].

In the current study, we dissected the roles of individual laminin-binding integrins in cellular processes that contribute to angiogenesis. We employed RNAi technology together with organotypic angiogenesis assays—which model angiogenesis in an ECM environment, similar to that present during tissue repair [24,25,26]—in addition to transwell migration assays. Using these assays, we previously demonstrated that endothelial cells secrete both laminin-411 and laminin-511 during the formation of endothelial tubes [7]. We also showed that the expression of these laminin isoforms, as well as α6 integrins, are required for these processes [7]. In addition, we implicated the α6-dependent regulation of the angiogenesis-associated gene angiopoitin-2 (ANGPT2) and the chemokine receptor, CXCR4 [7]. However, our previous studies did not distinguish contributions from α6β1 or α6β4 or whether the laminin-binding integrin α3β1 also contributes to endothelial morphogenesis in organotypic angiogenesis assays. Our current data suggest that the α3β1 and α6β4 integrins contribute to endothelial sprouting by non-redundant mechanisms. Moreover, gene expression analyses indicate that these integrins contribute to the expression of distinct but overlapping sets of angiogenesis-associated genes. To our knowledge, our study is the first to examine the contribution of laminin-binding integrins to the regulation of a set of previously identified angiogenesis-associated genes.

## 2. Materials and Methods

### 2.1. Immunofluorescence Microscopy

#### 2.1.1. Immunostaining

Murine tissue: Murine ears were separated into dorsal and ventral layers and the epidermis subsequently removed with the blunt end of a pair of forceps in EDTA. Whole-mount murine retinas, ears and cryo-sectioned skin were fixed with 4% PFA for 30 min, permeabilized with 0.5% Triton X-100 in PBS for 30 min, and then blocked with 2% BSA and 0.1% saponin in PBS overnight at 4 °C. Antibodies were diluted in 2% BSA and 0.1% saponin in PBS and incubated with tissue for 48 h at 4 °C. Samples were then washed 4× with 0.1% saponin in PBS at RT over the course of 24 h, then incubated overnight with the appropriate secondary antibodies (1:1000 dilution) at 4 °C. Following secondary antibody staining, samples were washed 4× with 0.1% saponin in PBS at RT over the course of 24 h and mounted with SlowFade Gold antifade reagent (ThermoFisher, Waltham, MA, USA). See Table S1 for antibody information. Bead-sprout and planar co-culture assays: Bead-sprout and planar co-cultures were fixed with 4% PFA (Electron Microscopy Sciences, Hatfield, PA, USA) for 15 min, permeabilized with 0.5% Triton X-100 in PBS for 15 min, and then blocked with 2% BSA in PBST (PBS + 0.1% Tween20) for 1 h at RT. In the case of bead-sprout assays, fibroblasts were removed using trypsin-EDTA Solution 10× (59418C, Sigma Aldrich, St. Louis, MO, USA) prior to fixation and imaging. Antibodies were diluted in 2% BSA in PBST, and incubated with cells overnight at 4 °C. Samples were then washed 4× with PBST at RT over the course of 4 h, then incubated for 1 h with the appropriate secondary antibodies (1:1000 dilution). Following secondary antibody staining, samples were washed 3× with PBST at RT for 1 h and mounted with SlowFade Gold antifade reagent (ThermoFisher). See Table S1 for antibody information.

#### 2.1.2. Microscopy

Most samples were analyzed using a Nikon inverted TE2000-E microscope equipped with phase contrast and epifluorescence, a digital CoolSNAP HQ camera, a Prior ProScanII motorized stage, and a Nikon C1 confocal system, using EZC1-3.90 and NIS-Elements -AR acquisition software (Nikon, Melville, NY, USA). Images were acquired with Plan Fluor 4×/0.13, Plan Fluor 10×/0.30, Plan Fluor ELWD 20×/0.45, Plan Apo 40×/1.0 oil, and Plan Apo 100×/1.4 oil objectives, and analyzed with NIS elements (Nikon). Contrast and/or brightness were adjusted for some images to assist in visualization. To image murine skin at high resolution, a Zeiss LSM 880 confocal microscope system with AiryScan detector and a FAST Airyscan module mounted on an AxioObserver (Carl Zeiss, Inc, Peabody, MA, USA)was used. The LSM 880 confocal detection system had 34 spectral-detection channels consisting of a cooled 32 element GaAsP detector array with two flanking photomultiplier tubes (PMTs). Wavelength separation was achieved using high-efficiency grating; maximum spectral resolution was 3 nm over a 286 nm range (410 to 696 nm). Objective lenses: 10×|0.45 NA (air), 20×/0.8 NA DIC, 25×|0.8 multi-immersion DIC, 40×|1.4 NA W DIC, 63×|1.4 NA DIC oil, C-Apochromat 40×/1.2 W/korr FCS, Plan-Apochromat 63×/1.4 NA oil DIC ELYRA. Six single-photon-excitation laser lines were available: 405, 458, 488, 514, 561 and 633 nm.

#### 2.1.3. Analysis of Sprouting

Sprout lengths in bead-sprout assays were measured by tracing each sprout using NIS elements (Nikon), and sprouts per bead were counted manually.

### 2.2. Cell Culture

Human umbilical vein endothelial cells (HUVECs) were from Lonza (Allendale, NJ, USA) and were cultured in in EGM-2 (Lonza, Walkersville, MD, USA, #CC-3162), and used between passages 2–6. Adult human dermal fibroblasts (HDFs) were isolated and characterized as previously described [27,28]; they were generously provided by Dr. Livingston Van De Water (Albany Medical College, Albany, NY, USA) and used between passages 8–14. Human embryonic kidney epithelial 293FT cells (HEK293FT) were a kind gift from Dr. Alejandro Pablo Adam (Albany Medical College). HDFs and HEK293FT cells were cultured in DMEM (Sigma Aldrich #D6429) containing 10% FBS (Atlanta Biologicals, Flowery Branch, GA, USA), 100 units/mL penicillin (Life Technologies, Carlsbad, CA, USA), 100 µg/mL streptomycin (Life Technologies, Carlsbad, CA, USA), and 2.92 µg/mL L-glutamine (GE LifeSciences, Marlborough, MA, USA). All cells were cultured at 37 °C in 5% CO_2_.

### 2.3. Organotypic Culture Assays

#### 2.3.1. Bead-Sprout Assay

To study endothelial sprouting, we employed the bead-sprout assay as described by Nakatsu and Hughes [26]. Cytodex 3 beads (GE) were coated at ~1000 HUVECs per bead inside of a 2 mL microcentrifuge tube for 4 h at 37 °C, mixed gently by inverting the tubes every 20 min, transferred to a T25 flask and incubated at 37 °C, overnight. Beads were then washed 3× with EGM-2 medium and re-suspended in PBS containing 3 mg/mL of fibrinogen (Sigma Aldrich, #F8630) and 0.15 U/mL of aprotinin (Sigma, #A6279). Thrombin (Sigma, #T4648) was added at a final concentration of 0.125 U/mL and the mixture was plated in wells of an 8-well slide (Corning, Corning, NY, USA #3-35411). The mixture was allowed to clot for 30 min at 37 °C. HDFs were then added to the top surface of the fibrin gel in EGM-2 medium at a concentration of 30,000 cells per well. The formation of sprouts and sprout lengths were assayed by either immunofluorescence or phase–contrast microscopy.

#### 2.3.2. Planar Co-Culture

As one organotypic culture, we utilized the planar co-culture model developed by Bishop and colleagues [24] and modified by the Pumiglia laboratory [25]. This model reconstitutes some of the complex interactions that occur during angiogenesis among endothelial cells, the ECM and supporting cells. To set up the co-culture, HDFs were seeded in tissue culture dishes with or without glass coverslips and cultured to confluence. The medium was changed to EGM-2. HUVECs were then seeded 16 h later at a density of 20,000 cells per 9 cm^2^ and cultured up to 10 days. Endothelial morphogenesis and α6β4 expression were analyzed by immunofluorescence microscopy.

### 2.4. siRNA

HUVECs were plated in 6-well tissue-culture plates and transfected with siRNA at a 50 nM concentration with RNAiMAX (ThermoFisher) using the protocol provided by the manufacturer. HUVECs transfected with siRNA were assayed for knockdown and used in migration assays 48 h after transfection. For bead-sprout assays (described above) HUVECs were transfected with siRNA during bead coating and assayed for knockdown at the end of experiment. Sources and nucleotide sequences of siRNAs used in this study are provide in Table S2.

### 2.5. Inducible shRNA

Doxycycline-inducible lentiviral (SMART) vectors harboring shRNAs targeting the α3 integrin subunit or a non-targeting (NT) shRNA were purchased from Dharmacon (Lafayette, CO, USA). Lentiviruses were produced by co-transfection of HEK293FT cells with the shRNA expression vector together with the packaging plasmid, psPAX2, coding for Gag, Pol, Rev, Tat (#12260, Addgene, Watertown, MA, USA); and the envelope plasmid, pMD2.G, coding for VSV-G (#12259, Addgene, Watertown, MA, USA). HUVECs were transduced with filtered viral supernatant plus 8 µg/mL polybrene. Cells were induced with doxycycline (100 ng/mL) for 48 h to insure efficient knockdown of α3 expression. Nucleotide sequences of shRNAs used in this study are provided in Table S2.

### 2.6. Quantitative PCR (qPCR)

TRIzol (ThermoFisher) was used to isolate RNA from siRNA transfected HUVECs, as well as HUVECs expressing shRNAs. Extraction of RNA from bead-sprout assays (described above) using TRIzol was performed after the removal of HDFs with trypsin-EDTA Solution 10× (Sigma #59418C). cDNA was synthesized with iScript Reverse Transcription Supermix (BioRad, Hercules, CA, USA) using 1 µg of RNA. Equal amounts of cDNA were used in qPCR reactions performed with iQ SYBR Green Supermix (BioRad, Hercules, CA, USA). PCR primers were pre-designed by, and purchased from, Sigma Aldrich or Integrated DNA Technologies, as indicated in Table S3, together with their nucleotide sequences.

### 2.7. Western Blotting

Western blotting was used to confirm RNAi-induced knockdown. Cells were lysed in mRIPA buffer (50 mM Tris pH 7.4, 1% NP-40, 0.25% Na Deoxycholate, 150 mM NaCl, 1 mM EDTA) containing both phosphatase (Sigma, #4906837001) and protease inhibitor cocktails (ThermoFisher, 78440, Waltham, MA, USA). Equal amounts of protein (20 to 40 µg) were separated by SDS-PAGE and transferred to nitrocellulose for antibody probing. Imaging was performed with a ChemiDoc XRS+ (BioRad) and quantitation with Image Lab (BioRad). See Table S1 for antibody information.

### 2.8. Migration

HUVECs were used in migration assays 48 h post-transfection with siRNA, or 48 h post-treatment with doxycycline (100 ng/mL) to induce the expression of SMART Vector shRNAs. After overnight culture in serum-free EGM-2 medium, fifty thousand cells were seeded, in triplicate, into three wells of two separate 24-well plates, one with and without transwells. Serum-containing EGM-2 was then added to the lower chamber. Following an incubation for 4 h at 37 °C in 5% CO_2_, transwells were fixed with 4% PFA (Electron Microscopy Sciences). Cells that had not migrated though the filter were gently removed with cotton swabs before staining with DAPI. The lower membrane was imaged with a 4× objective and density quantified using ImageJ (NIH). Cell seeding efficiency was determined by performing toluidine blue assays in the 24-well plates. These assays were performed by fixing cells with 70% ethanol at room temperature for 1 h, followed by a wash with dH_2_O and staining with 0.05% toluidine blue at room temperature for an additional 2 h. After the wash with dH_2_O, toluidine blue was extracted with 10% acetic acid at 0.3 mL/well and absorbance measured at 650 nm, using 405 nm as reference on a Synergy2 microplate reader (BioTek Instruments, Winooski, VT, USA). An empty well was processed the same way and used for baseline. Migration efficiency was determined by dividing DAPI density by absorbance.

### 2.9. Animal Experiments

All animal experiments and procedures were performed in accordance with the Albany Medical College Institutional Animal Care and Use Committee (IACUC) regulations; they were performed in accordance with protocols 18-05003 and 18-07001, approved by the Albany Medical College IACUC. Adult murine retinas and ears were harvested from both male and female C57BL/6 mice between the ages of 8–12 weeks. Adult murine skins were harvested from male and female C57BL/6 mice expressing the transgenes: LSL-tdTomato and VE-Cadherin-driven CreERT2. Cre recombinase activity was induced via oral gavage of 10 µg/g of tamoxifen per day for 5 days.

### 2.10. Statistical Analysis

Statistical analysis was performed with GraphPad Prism software using Single-Sample *t*-test or one-way ANOVA, with post-hoc analysis using Dunnett’s or Tukey’s multiple comparisons tests as indicated in the figure legends. A *p*-value of *p* < 0.05 was considered to be statistically significant.

## 3. Results

### 3.1. Integrin α6β4 Is Expressed in Veins and Small Vessels of the Dermis

**Figure 1:**
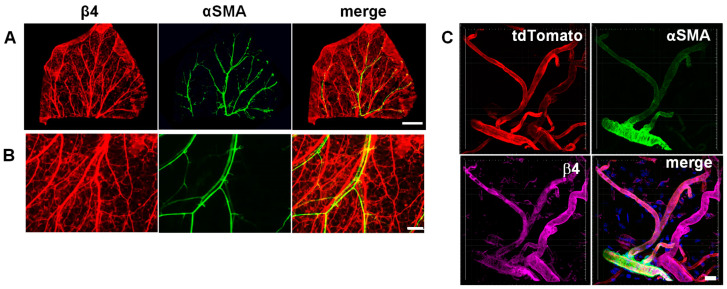
The α6β4 integrin is expressed in veins and venules of the dermal vasculature: (**A**) immunofluorescence staining of whole-mount mouse ear for α-SMA and β4-integrin subunit. Scale bar = 2 mm; (**B**) image of β4 and α-SMA expression acquired at a higher magnification. Scale bar = 500 μm; (**C**) confocal immunofluorescence staining of mouse skin for α-smooth-muscle actin and β4-integrin subunit. Vasculature is identified by tdTomato (red) expression. Image is a maximum projection of acquired z-stack. Arrowhead indicates the location of an arteriole. Scale bar = 20 μm.

Previous studies by us and others showed the expression of α6β4 in tumor-associated angiogenic vessels, as well as angiogenic vessels associated with dermal wound repair [22,23]. In contrast, others reported that α6β4 expression is restricted to arterioles [20]. More recently, single-cell RNA sequencing (scRNA-seq) data of vascular cells of the mouse brain and lung indicated that in the brain, β4 mRNA is expressed at higher levels in arterial endothelial cells compared to venous and capillary endothelial cells. In contrast in the lung vasculature, β4 mRNA was more widely expressed, including in veins and venules, with higher expression in capillary endothelial cells [29]. The recent scRNAseq data motivated us to re-examine the protein expression of α6β4 to determine whether it is expressed by venous endothelial cells, since angiogenesis is associated with the sprouting of vessels from existing venules and capillaries. Since the β4 subunit only dimerizes with the α6 subunit [30], analyzing the expression of the β4 subunit is a reliable reporter for the expression of α6β4. We examined the expression of α6β4 in vasculatures of the dermis and brain (retinal vasculature) by immunofluorescence microscopy. Consistent with the scRNAseq data, α6β4 was more widely expressed in endothelial cells outside the brain, specifically in the dermis; this includes in small vessels and venules, in addition to some endothelial cells that are positive for α-SMA (Figure 1A–C). In the adult murine retinal vasculature, which is considered part of the brain vasculature, α6β4 expression is colocalized with strong expression of α-SMA, which is consistent with its expression in arteries/arterioles in the brain (Figure S1). Thus, our results examining the expression α6β4 protein level are consistent with the previously published scRNA-seq results documenting β4 RNA expression in endothelial cells from different parts of the vasculature.

### 3.2. Integrin α6β4 Promotes Endothelial Morphogenesis

**Figure 2:**
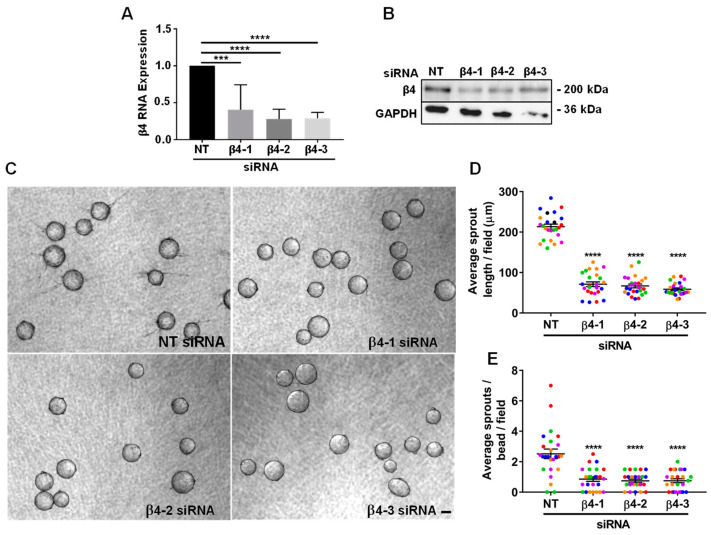
Integrin α6β4 regulates endothelial morphogenesis: (**A**) The efficiency of β4 depletion using 3 siRNA targeting sequences was determined by qPCR. Data were normalized to β-actin and then to non-targeting (NT) control. Plotted is the mean RNA expression ± s.d. *n* = 5 independent experiments; (**B**) representative western blot showing the efficiency of β4 depletion at the protein level. Endothelial cells from the same batches of endothelial cells depleted of the β4 subunit used in experiments presented in Figure 2 were employed in bead-sprout assays; (**C**) shown are representative images of 6-day bead sprouts transfected with non-targeting and β4-targeting siRNA sequences 1–3. Scale bar = 100 μm; (**D**,**E**) depletion of α6β4 expression inhibits sprouting. Quantitation of sprout length (top) and number of sprouts (bottom) from 6–8 beads in each of five randomly selected fields in five independent experiments; (**D**) plotted is the average sprout length per field ± s.e.m. *n* = 25 fields; (**E**) quantitation of the average sprouts per bead per field ± s.e.m. Each independent experiment is represented by a distinct color. Data were analyzed using two-tailed Student’s *t*-test. Each independent experiment is represented by a distinct color. *** *p* ≤ 0.001, **** *p* ≤ 0.0001.

Previous studies have suggested a role for α6β4 in vessel maturation and stability, and its expression has been both positively and negatively associated with angiogenesis [21,22,23,31]. To determine whether α6β4 expression contributed to processes associated with angiogenesis, we employed an organotypic angiogenesis model (also referred to as a bead-sprout assay), in which cytodex beads coated with endothelial cells (HUVECs) are place in a fibrin gel overlayed with a confluent layer of dermal fibroblasts, which provide important paracrine signals together with VEGF to promote endothelial sprouting into the gel and subsequent formation of tubes from these sprouts [26,32,33]. Using this model, we previously demonstrated that the expression of α6 integrins is required to promote endothelial sprouting and tube formation; however, we did not determine the specific contributions of α6β1 and α6β4. Since HUVECs express α6β4 on their cell surface (Figure S2), we used the same organotypic co-culture approaches to determine the contribution of α6β4. We analyzed 6-day sprouts of endothelial cells depleted of β4, compared to the control. The efficiency of β4 mRNA and protein depletion with three distinct targeting siRNAs is shown in Figure 2A,B. Each of the β4-targeting siRNAs significantly inhibited sprouting, as shown in the representative images of 6-day bead sprouts in Figure 2C. Knockdown of the β4 subunit, and thus α6β4, with each of these three siRNA sequences resulted in a significant decrease in both sprout length (Figure 2D) and the number of sprouts per bead (Figure 2E). Thus, α6β4 contributes to endothelial sprouting in this organotypic assay.

### 3.3. Integrin α6β4 Promotes Endothelial Migration

**Figure 3:**
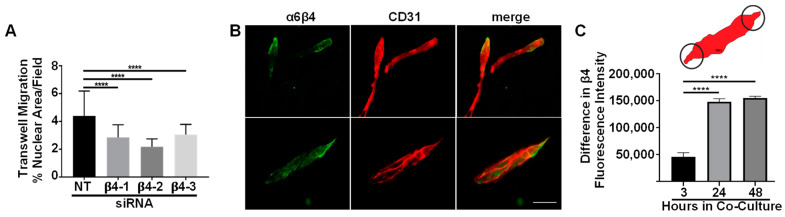
Depletion of α6β4 reduces endothelial cell migration: (**A**) Analysis of nuclear staining in transwell migration assays with non-targeting (NT) or β4-targeting cells. Fifteen fields were analyzed from each of two independent experiments. Plotted is the mean percentage of area per field stained for nuclei ± s.d. *n* = 30. Data were analyzed by one-way ANOVA and Dunnett’s multiple comparisons test; (**B**,**C**) the polarized expression of β4 in endothelial cells was analyzed at 3, 24, and 48 h; (**B**) representative images of endothelial cells at 24 h immunostained for the β4 integrin subunit (green) and CD31 (red). Scale bar = 100 µm; (**C**) plotted is the mean difference in β4/CD31 fluorescence intensity between polar ends of endothelial cells or cell cords using a constant ROI (see top panel in C for diagram) ± s.e.m. Fifteen fields were analyzed from three independent experiments (*n* = 3 independent experiments). Data were analyzed by one-way ANOVA and the Tukey’s multiple comparisons test. **** *p* ≤ 0.0001.

Since cell motility is an important aspect of angiogenesis, we examined the role of α6β4 in endothelial cell migration. Although we previously demonstrated that α6 integrins promote endothelial migration, we did not determine whether α6β4 contributed to the regulation of cell migration by α6 integrins [7]. We inhibited the expression of α6β4 using RNAi technology. β4-depleted endothelial cells did not exhibit defects in proliferation or survival in 2D culture (data not shown), which is consistent with β4-depleted endothelial cells in vivo [20,22,23]. We inhibited the expression of α6β4 of by targeting the β4 subunit with three distinct siRNA-targeting sequences, and measured the effect in transwell migration assays. The depletion of β4 resulted in a significant reduction in cell migration across gelatin-coated filters (Figure 3A). Importantly, this phenotype was consistent across all three siRNA-targeting sequences, and consistent with previous studies that suggested a role for α6β4 in the regulation of endothelial cell migration [22]. Although α6β4 does not bind to gelatin, our previous studies demonstrated that these endothelial cells secrete their own laminin substrates. Thus, endothelial cells are similar to epithelial cells, which secrete their own laminin for migration [34,35,36].

The α6β4 integrin is polarized to the leading edge of migrating epithelial cells to promote their migration [37,38,39]. The α6β4 integrin has also been observed at the tips of sprouting vessels in vivo [40]. Thus, we were interested in examining the localization of α6β4 during the formation of endothelial tubes. In these experiments, we employed a second organotypic assay, referred to as the planar co-culture assay. In this assay, endothelial cells are plated at low density on a confluent layer of dermal fibroblasts, which become embedded in a dense fibronectin matrix. Endothelial cells attach and spread within 1 h and begin to elongate, migrate and form cords after approximately 24 h [7,41]. Therefore, we employed immunofluorescence microscopy to examine the cellular localization of endothelial α6β4 at 3, 24 and 48 h. Co-cultures were immunostained for the β4 subunit and the endothelial marker, CD31 (Figure 3B). This was feasible as fibroblasts that are present in the co-culture do not express either α6β4 or CD31. To measure the polarized distribution of α6β4 in endothelial cells/cords, we used CD31 to establish the endothelial area and determined the difference in β4 fluorescence intensity on either ends of endothelial cells or cords using a constant-sized ROI (Figure 3C, top panel). At 3 h of co-culture, individual endothelial cells are easily distinguishable. Little polarized localization was observed at this time (Figure 3C). At 24 and 48 h, when endothelial cords had begun to form cords [7,41], the localization of α6β4 was predominately stronger at one end of the endothelial structure (Figure 3C). Although we cannot discern whether the concentrated localization occurs at the migrating front, the polarized distribution of α6β4 is consistent with its role in endothelial migration and the localization of α6β4 in sprouting vessels in vivo.

### 3.4. Integrin α6β4 Promotes the Expression of ANGPT2 and Other Angiogenesis-Associated Genes

**Figure 4:**
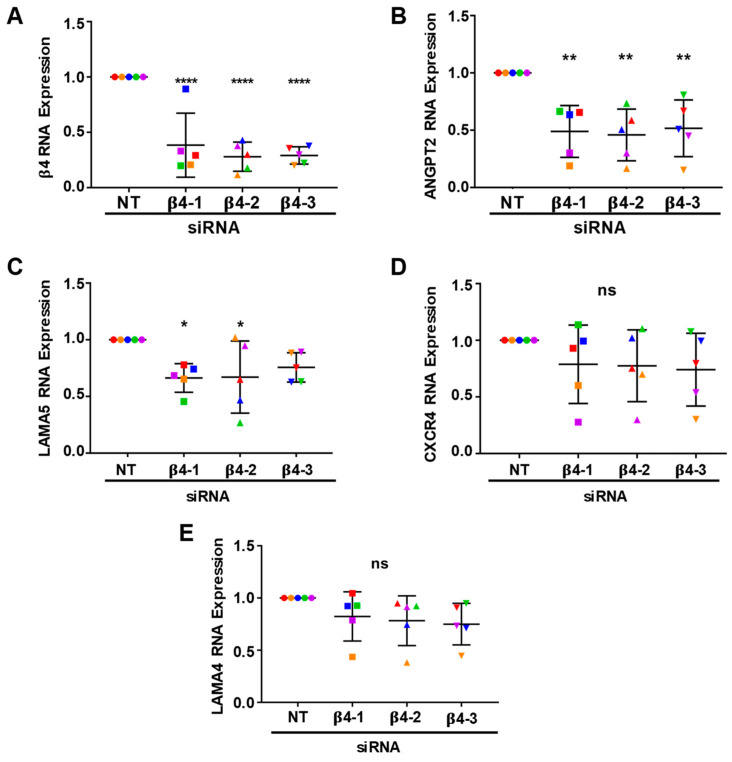
The expression ANGPT2 is positively regulated by integrin α6β4. RNA was isolated from 6-day bead sprouts and analyzed by qPCR: (**A**) Shown is the efficiency of β4 depletion using three distinct siRNA targeting sequences (also shown in Figure 2A) and the effects of β4 depletion on the expression of the (**B**) ANGPT2, (**C**) LAMA5, (**D**) CXCR4, and (**E**) LAMA4. Data were normalized to β-actin and then to the non-targeting (NT) control. Plotted is the mean RNA expression ± s.d. *n* = 5 independent experiments. Data were analyzed using a Single-Sample *t*-test. Each independent experiment is represented by a different color. ns = not significant, * *p* ≤ 0.05, ** *p* ≤ 0.01, **** *p* ≤ 0.0001.

Since we determined that α6β4 contributed to both cell migration and endothelial sprouting, we next sought to determine whether it also contributed to the regulation of the angiogenesis-associated genes that we previously identified to be inhibited in α6-depleted cells. Expression of the α5 chain of laminin (LAMA5) of laminin-511, CXCR4, and ANGPT2 mRNA transcripts were previously determined to be positively associated with the expression of endothelial α6 integrins [7]. To evaluate the contribution of α6β4 to the expression of these genes, we isolated RNA from endothelial cells treated with non-targeting and β4-targeting siRNA from 6-day bead-sprout assays (shown in Figure 3) and analyzed gene expression by qPCR (Figure 4). The expression of β4 mRNA was significantly reduced by all three β4-targeting siRNAs (Figure 4A). Interestingly, a significant decrease in ANGPT2 expression was also observed with all three β4-targeting siRNAs (Figure 4B), indicating that α6β4 contributes to the regulation of ANGPT2 mRNA expression. The expression of LAMA5 was significantly decreased in cells treated with either of the two β4-targeting siRNAs (Figure 4C), while trending downward in cells treated with a third siRNA. These data suggest that α6β4 promotes the expression of LAMA5 mRNA. Notably, the expression of CXCR4 was not inhibited in β4-depleted endothelial cells (Figure 4D), suggesting that α6β1 is likely to be responsible for the previously described regulation of CXCR4 by α6 integrins [7]. Similar to our published results for α6-depleted endothelial cells, the expression of the α4 chain (LAMA4) of laminin-411 was not affected in β4-depleted cells (Figure 4E).

**Figure 5:**
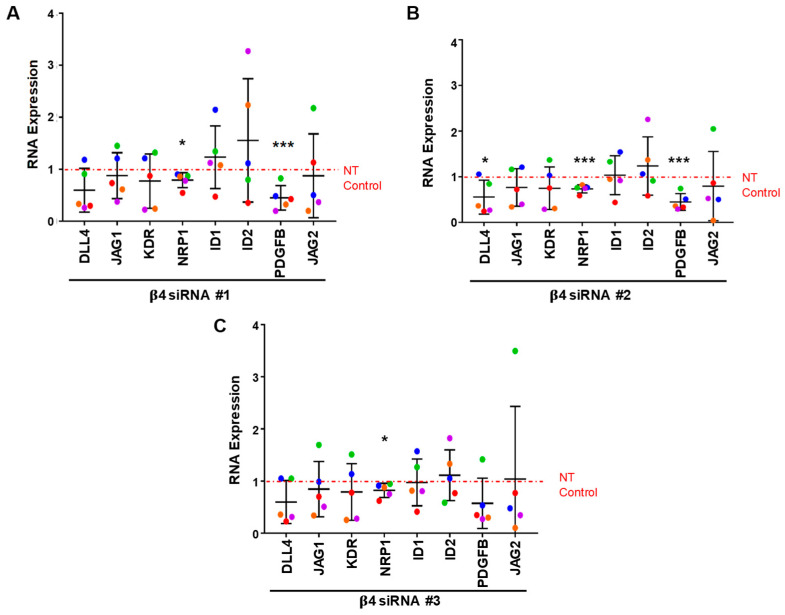
The expression of NRP1 and PDGFB are positively associated with integrin α6β4 expression. Changes in RNA expression in 5-day bead-sprout assays of β4-depleted endothelial cells and control cells were measured by qPCR. The efficiency of β4 depletion using 3 siRNA targeting sequences was previously shown in Figure 4. Effects of β4 knockdown with siRNA #1 (**A**), siRNA #2 (**B**), or siRNA# 3 (**C**) on the expression of genes associated with angiogenesis. Data were normalized to β-actin and then to non-targeting (NT) control. Plotted is the mean RNA expression ± s.d. *n* = 5 independent experiments. Mean expression of each gene was compared to non-targeting (NT) control. Data were analyzed using a Single-Sample *t*-test. Each independent experiment is represented by a different color. * *p* ≤ 0.05, *** *p* ≤ 0.001.

To determine whether the depletion of α6β4 integrin affected the expression of other angiogenesis-associated genes [42,43,44], we analyzed the expression of Delta-like ligand 4 (DLL4), Jagged-1 (JAG1), Jagged-2 (JAG2), vascular endothelial growth factor receptor 2 (KDR), neurophilin-1 (NRP1), inhibitor of DNA-binding 1 (ID1), inhibitor of DNA-binding 2 (ID2), and Platelet-derived growth factor β (PDGFB) genes by qPCR (Figure 5A–C). The results indicate that the expression of NRP1 mRNA was significantly inhibited by all three β4-targeting siRNAs. Additionally, a significant downregulation of PDGFB mRNA was observed with two of the three β4-targeting siRNAs, with the third siRNA exhibiting a downward trend (Figure 5A,B). Taken together, these results suggest that α6β4 contributes to positive regulation of the expression of ANGPT2 and NRP1 RNA transcripts, and it is likely to promote the expression of PDGFB and LAMA5 mRNAs as well.

### 3.5. Integrin α3β1 Plays Overlapping and Distinct Roles during Endothelial Morphogenesis

**Figure 6:**
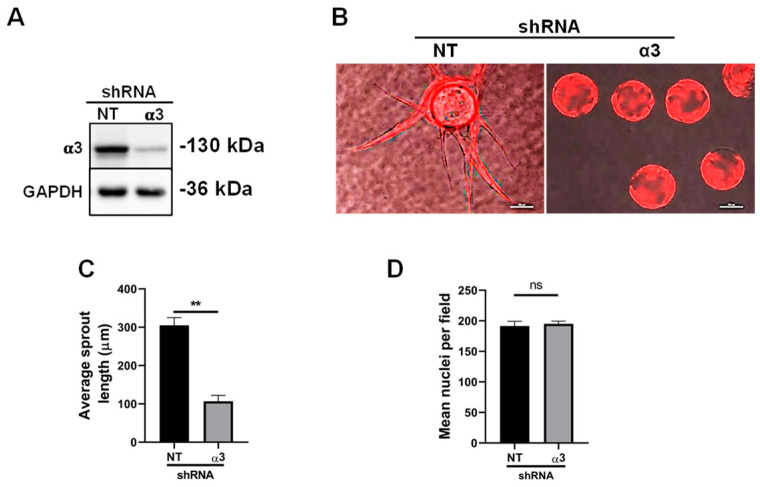
Depletion of endothelial integrin α3β1 inhibits early endothelial morphogenesis but does not affect endothelial migration: (**A**) representative western blot of the expression of the α3 integrin subunit after induction of α3-targeting or non-targeting (NT) shRNA; (**B**) representative images of 6-day bead-sprout assays. Shown are overlays of phase contrast and fluorescent images of the same fields of endothelial cells expressing RFP in conjunction with shRNA under doxycycline regulation. Scale bar = 100 µm; (**C**) plotted is the mean sprout length measured from 3 independent experiments of 6-day bead-sprout assays with 10 randomly selected fields each, averaging 6–8 beads per field ± s.e.m. *n* = 3 independent experiments; (**D**) analysis of nuclear staining in transwell migration assays with non-targeting (NT) or α3-targeting cells. Five fields were analyzed from each of three independent experiments. Plotted is the mean area of nuclei per field, normalized to number of cells seeded ± s.e.m. *n* = 3 independent experiments. Data were analyzed by two-tailed Student’s *t*-test. ns = not significant, ** *p* ≤ 0.01.

In addition to α6β1 and α6β4, endothelial cells also express integrin α3β1 to engage their laminin substrates [10,45]. Since the expression of α3β1 was found to be a negative regulator of angiogenesis in vivo [14], we questioned whether it served a similar role in our organotypic cultures. To determine whether the depletion of α3β1 enhanced endothelial morphogenesis, we employed lentiviral vectors for the doxycycline inducible expression of either non-targeting (NT) or α3-targeting shRNA that was accompanied by the expression of an RFP reporter. The induction of the expression of α3-targeting shRNA significantly inhibited the expression of the α3 subunit, and thus, the α3β1 integrin (Figure 6A). Notably, no effects on proliferation or survival were observed in 2D culture, consistent with α3-depleted endothelial cells in vivo [15]. The efficient depletion of the α3 subunit from endothelial cells inhibited their ability to form sprouts in bead-sprout assays (Figure 6B,C). Images of 6-day bead-sprout assays with endothelial cells expressing either NT or α3-targeting shRNA, together with RFP, are shown in Figure 6B. The quantification of three independent experiments is shown in Figure 6C. It is noteworthy that sprouting was severely inhibited in α3-depleted cells. However, unlike the depletion of α6-integrins, depletion of α3β1 did not impact cell migration across gelatin-coated transwells (Figure 6D).

### 3.6. Integrin α3β1 Contributes to the Expression of a Distinct Set of Angiogenesis-Associated Genes

**Figure 7:**
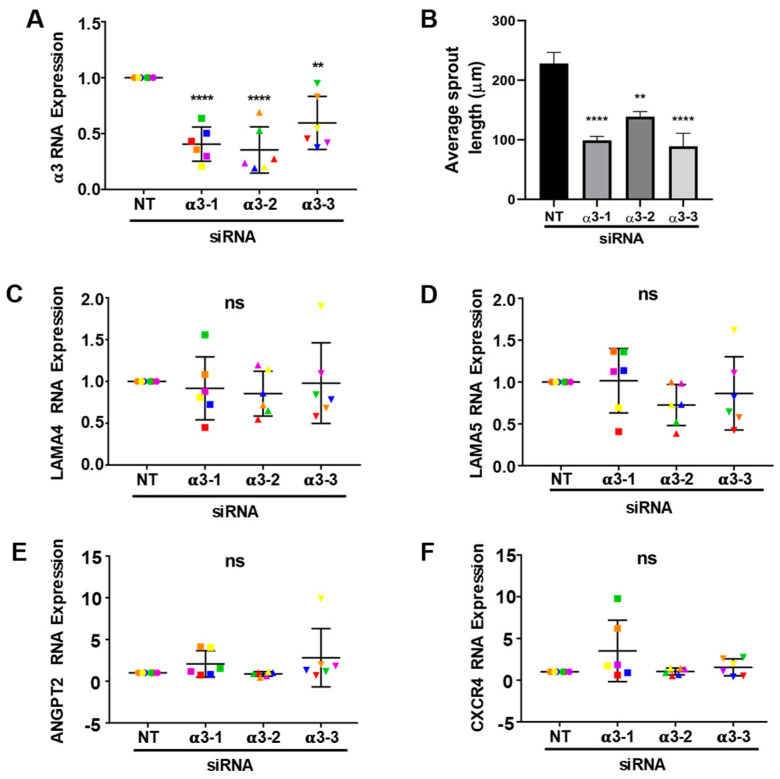
Integrin α3β1 expression is not required for the expression of CXCR4, ANGPT2, LAMA4, and LAMA5. (**A**) The efficiency of α3 depletion using 3 siRNA-targeting sequences was determined by qPCR from 6 independent experiments. Data were normalized to β-actin and then to non-targeting (NT) control. Plotted is the mean RNA expression ± s.d. *n* = 6 independent experiments. Data were analyzed by Single-Sample *t*-test; (**B**) quantitation of sprout length from 6–8 beads in each of 5 randomly selected fields in 6 independent experiments. Data plotted as the mean sprout length ± s.e.m. *n* = 6 independent experiments. Data were analyzed by one-way ANOVA with Dunnett’s multiple comparisons test; (**C**–**F**) the effects of α3 depletion on the expression of the LAMA5, LAMA4, ANGPT2, and CXCR4. Data were normalized to β-actin and then to non-targeting (NT) control. Plotted is the mean RNA expression ± s.d. *n* = 6 independent experiments. Data were analyzed by Single-Sample *t*-test. Each independent experiment is represented by a distinct color. ns = not significant, ** *p* ≤ 0.01, **** *p* ≤ 0.0001.

**Figure 8:**
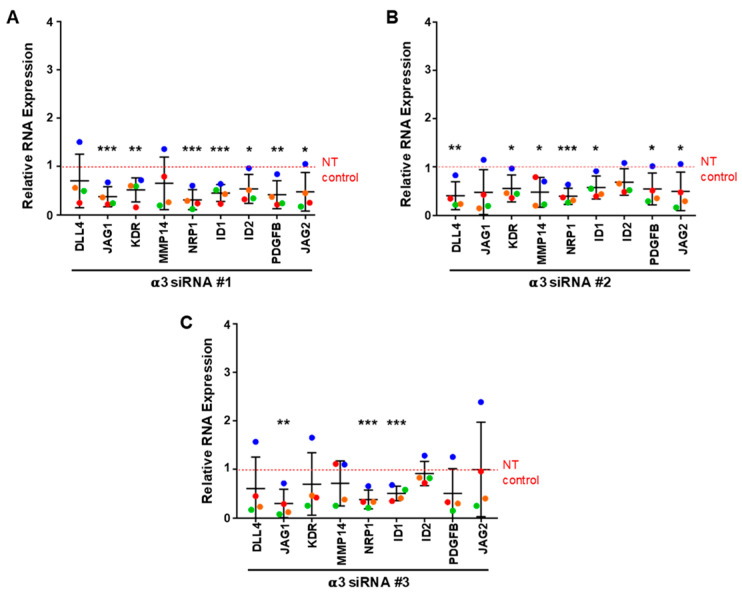
Regulation of angiogenesis-associated genes by integrin α3β1. Changes in RNA expression in 5-day bead-sprout assays of α3-depleted endothelial cells and control were measured by qPCR. The efficiency of α3 depletion using 3 siRNA targeting sequences previously shown in Figure 7. Effects of α3 knockdown with siRNA #1 (**A**), siRNA #2 (**B**), or siRNA# 3 (**C**) on the expression of genes associated with angiogenesis. Data were normalized to β-actin and then to non-targeting (NT) control. Plotted is the mean RNA expression ± s.d. *n* = 4 independent experiments. Mean expression of each gene was compared to non-targeting (NT) control. Data were analyzed using a Single-Sample *t*-test. * *p* ≤ 0.05, ** *p* ≤ 0.01, *** *p* ≤ 0.001.

Since depletion of α3β1 from endothelial cells also inhibited endothelial morphogenesis, we asked whether any of the genes that are regulated by α6 integrins (α6β1 and/or α6β4) are also regulated by α3β1. For these gene expression studies, we depleted the α3 integrin subunit using siRNAs with three distinct targeting sequences. All three efficiently inhibited expression of the α3 subunit gene, as shown by qPCR analysis (Figure 7A). As a control, we asked whether depleting the cells of α3β1 using siRNAs lead to defects in endothelial sprouting similar to the phenotype observed when α3 was depleted by shRNA. All three siRNAs resulted in a similar inhibition of endothelial morphogenesis (Figure 7B). Since our previous studies implicated α6 integrins in regulating the expression of LAMA5, ANGPT2, and CXCR4, but not LAMA4, we first examined the expression of these genes in α3-depleted cells. RNA was harvested from α3-depleted and control endothelial cells from 6-day bead-sprout assays and analyzed by qPCR. This analysis did not reveal significant changes in LAMA4, LAMA5, ANGPT2 or CXCR4 expression (Figure 7C–F). Expanded qPCR analysis for other angiogenesis-associated genes identified a significant downregulation of NRP1 and ID1 with all three α3-targeting siRNA sequences. A significant decrease in the expression of PDGFB mRNA was observed with two of the α3-targeting sequences, with the third showing a downward trend (Figure 8A–C). These results suggest that α3β1 regulates endothelial morphogenesis by distinct mechanisms, and that the loss of its expression cannot be compensated by α6β1 or α6β4. Notably, α3β1 and α6β4 regulated the expression of distinct but overlapping sets of angiogenesis-associated genes.

## 4. Discussion

**Figure 9:**
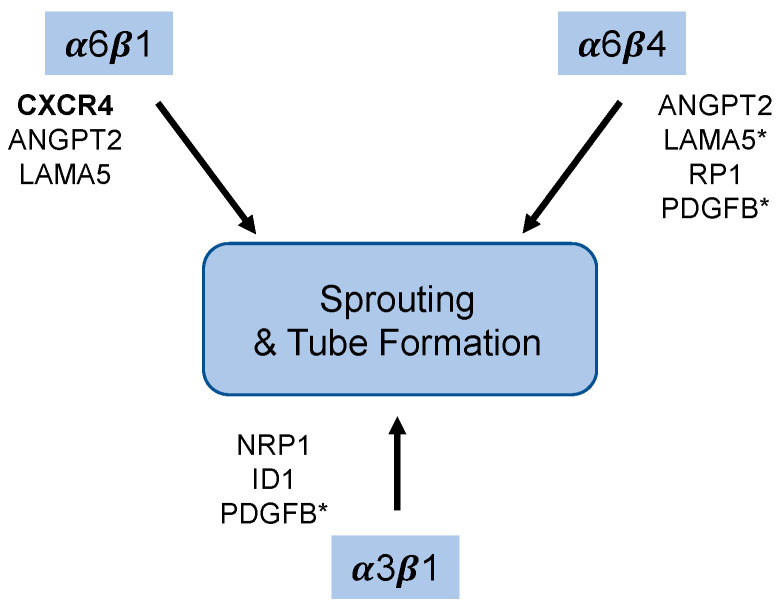
Schematic summary. RNAi-dependent depletion of the α3β1 or the α6 integrins, α6β1 and α6β4 inhibits endothelial sprouting and tube formation in organotypic co-culture models. The expression of CXCR4 shown in bold was inhibited only by the depletion of the integrin α6 subunit, and not by depletion of either the β4 or α3 subunit, suggesting that α6β1 is the only laminin-binding integrin that impacts CXCR4 expression (Figure 4 and Figure 7 and [7]). Depletion of either the α6 or β4 subunit inhibited migration, as well as the expression of ANGPT2 and LAMA5 mRNA; this suggests that α6β4 regulates the expression of these genes, but does not exclude the possibility of a contribution from α6β1. The depletion of α3β1 or α6β4 inhibited the expression of NRP1 and PDGFB, whereas the expression of ID1 was only inhibited by the depletion of α3β1. * Indicate genes whose expression was significantly inhibited by two of the three siRNA-targeting sequences.

Taken together, our data suggest that endothelial laminin-binding integrins play overlapping and distinct roles during endothelial morphogenesis (Figure 9). Our previous studies indicated that the expression of integrin α3β1 does not compensate for the depletion of α6 integrins in our assays [7]. Our current findings demonstrate that inhibiting the expression of α3β1 also inhibits morphogenesis, indicating that α6 integrins do not compensate for the loss of α3β1. Because the integrin β1 subunit dimerizes with multiple α subunits, we were unable to directly identify specific roles for α6β1. However, we did demonstrate that the α6β4 integrin plays an essential role in promoting endothelial sprouting, suggesting that both α6β1, and α6β4 may contribute to these processes. Transcriptome analysis indicates that α3β1, α6β1, and α6β4 regulate distinct sets of angiogenesis-associated genes at the level of mRNA expression (see Table S4). For example, our previous studies indicated that depletion of the α6 subunit inhibited the expression of both ANGPT2 and CXCR4; however, we did not determine whether α6β1 and/or α6β4 was responsible for this regulation [7]. Here, we show that depletion of α6β4 resulted in the inhibition of the expression of ANGPT2 mRNA, but did not alter the expression of CXCR4. While the expression of α6β4 is positively correlated with ANGRT2 mRNA expression, we are not excluding the possibility that α6β1 may also contribute to ANGPT2 expression. Our data also indicate that α6β4 does not significantly contribute the expression of CXCR4 mRNA; this suggests that α6β1 is responsible for regulating the expression of CXCR4 and that α6β1 regulates endothelial morphogenesis, at least in part without contribution from α6β4, as the expression of recombinant CXCR4 partially rescued endothelial morphogenesis when the α6 subunit was depleted [7]. Notably, the depletion of α3β1 did not alter the expression of ANGPT2 or CXCR4, supporting its distinct role in endothelial cells.

Others have shown that the overexpression of the α6 and β4 subunits in cultured endothelial cells promotes transwell migration on a laminin-332 substrate [22]. We demonstrated that endogenously expressed α6β4 positively regulates migration in cultured endothelial cells without the addition of a laminin substrate or the need for overexpression. Interestingly, our published studies demonstrated that the endothelial expression of laminin-511, a ligand for α6β4, is regulated by α6 integrins [7] (Figure 4C). Thus, α6-dependent regulation of laminin-511 may be involved in promoting migration by α6β4 as observed in our current studies (Figure 2C) and explain why α3β1 is not required for migration in our assays (Figure 6D and Figure 7D). It is important to note that others have demonstrated, in other cell types, that α6β4 can promote migration on non-laminin substrates, suggesting the possibility that α6β4 may regulate cell migration by a ligand-independent mechanism [46].

In epithelial cells, α6β4 becomes polarized at the leading edge of migrating epithelial cells to promote their migration [37,38,39]. We demonstrated that endothelial α6β4 also contributes to endothelial cell migration. We observed the polarized localization of α6β4 during the migratory phase of endothelial tube morphogenesis in planar co-culture, similar to the polarized endothelial expression of α6β4 at the tips of endothelial sprouts during neovascularization in human neonatal foreskins [40]. We had previously demonstrated that α6β4 associates with the vimentin intermediate filament by mechanisms that require the β4 subunit cytoplasmic domain [47,48]. Interestingly, recent studies demonstrated that α6β4 localizes with vimentin in puncta in lamellipodia at the leading edge, to promote the migration of epithelial cells [49]. Thus, the α6β4 integrin may similarly regulate migration in endothelial cells.

Sprouting angiogenesis requires the invasion of endothelial cells into the surrounding ECM [50]. The inhibition of sprouting by β4-depleted endothelial cells in our bead-sprout assays suggests that α6β4 may also promote invasion during angiogenesis. Notably, α6β4 has been shown to contribute to cancer cell invasion [37], indicating that the α6β4 integrin likely contributes to invasion in multiple cellular contexts. Importantly, others have identified a role for endothelial α6β1 integrins both in culture and in vivo in the formation of invasive structures known as podosome rosettes, which concentrate the membrane-associated protease, MT1-MMP [19]. The α6β1, but not the α6β4 integrin, was recruited to rosettes, at least when its localization was examined in cell culture [19], suggesting that α6β1 and α6β4 likely contribute to invasion by different mechanisms. It will be important to identify the mechanisms by which α6β4 contributes to endothelial invasion and how these differ from α6β1.

Our current study identified a role for α6β4 in the regulation of ANGPT2 mRNA, although we cannot exclude the possibility that α6β1 also contributes to this regulation. Angiopoietin-2 (product of the ANGPT2 gene) serves as an antagonist to Tie-signaling activated by angiopoietin-1 [42,50,51]. Since angiopoietin-1 is secreted by neighboring mural cells in vivo, it is unclear how the loss of angiopoietin-2 expression in our organotypic model inhibits morphogenesis. However, some studies have indicated that angiopoietin-2 can activate Tie-2 in an autocrine fashion, suggesting that angiopoietin-2 may have a cell-autonomous effect in promoting new vessel formation [52]. This possibility would be interesting to address in future studies.

Additionally, our results indicate that α6 integrins do not compensate for the loss of α3β1, as depletion of the α3 subunit also inhibited morphogenesis in bead-sprout assays, suggesting that α3β1 regulates these processes by distinct mechanisms. Consistent with this notion, the loss of α3β1 expression did not inhibit migration in our assays and affected the expression of a distinct set of angiogenesis-associated genes compared to those regulated by α6 integrins (Table S4). In vivo studies by da Silva and colleagues reported that endothelial α3β1 acted as a repressor of pathological angiogenesis, as its deletion enhanced tumor-associated angiogenesis through the upregulation of KDR (VEGFR2) [15]. We did not observe an upregulation of KDR/VEGFR2, at least at the transcript level; however, we did not test whether the translation of VEGFR2 was altered in our assay. It is important to note that although da Silva and colleagues demonstrated that the expression of α3β1 was absent from tumor-associated vessels, blood vessels in the surrounding normal skin were still positive for α3β1 [15]. This implies that endothelial α3β1 was present during the initiation of tumor-induced angiogenesis. Thus, this in vivo model may not be ideal to examine the role of α3β1 during the initial steps of angiogenesis, when endothelial cells first respond to angiogenic signals, and may explain the differences with our current findings.

Interestingly, we observed the downregulation of NRP1 and ID1 using all three α3 siRNA-targeting sequences. NRP1 is a critical protein enriched with tip cells; it functions as a co-receptor forVEGFR2 signaling [53], and contributes to angiogenesis, in part by promoting the formation of filopodia through the activation of CDC42 [54]. It will be interesting to determine whether the expression of recombinant NRP1 can rescue the defects in morphogenesis in cells depleted of α3β1. It is important to note that we also observed a downregulation of NRP1 expression using all three siRNAs targeting the β4 subunit, suggesting that α6β4 also contributes to the regulation of NRP1 expression. We did not observe the inhibition of NRP1 expression in α6-depleted cells, presumably because α6β4 is more efficiently depleted when siRNA targeting the β4 subunit is employed.

As indicated above, α3β1 promotes the expression of ID1, a transcriptional regulator, which plays a role in angiogenesis during embryogenesis and tumor formation, as well as during endothelial morphogenesis in cell culture models [55,56,57,58,59]. Further studies are needed to determine whether the downregulation of ID1 contributes to the observed phenotype in α3-depleted endothelial cells in our organotypic assays. It is important to note that the α3β1 integrin likely promotes angiogenesis by multiple mechanisms. For example, in endothelial cells, α3β1 forms a ternary complex with the tetraspanin CD151 and the membrane-anchored matrix metalloproteinase and MT1-MMP, to promote appropriate proteolysis; the loss of CD151 results in a dramatic loss of α3β1/MT1-MMP association [60]. These observations have significant implications, as CD151 has been shown to promote angiogenesis [61]. Interestingly, α3β1 has been associated with tumor-cell invasion [62] and has been shown to regulate MMP-9 RNA stability in keratinocytes [63,64]. Given the matrix-dense environment of organotypic assays employed in our studies, the positive regulation of proteases by endothelial α3β1 during morphogenesis could possibly explain the lack of tube formation and sprouting by α3-depleted endothelial cells.

In summary, our current studies demonstrate that the expression of α6β4 and α3β1 regulate endothelial sprouting in our organotypic angiogenesis assay by non-redundant mechanisms, and regulate the expression of distinct sets of angiogenesis-associated genes. It will be important to identify the molecular mechanisms involved. Future studies are needed to determine the signaling pathways downstream of integrins that regulate the expression of these genes, and how these genes functionally contribute to processes needed for angiogenesis.

## Data Availability

This study did not report any datasets to be publicly archived.

## Supplementary Materials

The following are available online at https://www.mdpi.com/article/10.3390/cells11050816/s1, Figure S1: Integrin α6β4 is primarily expressed in the arterial vessels of adult mouse retina; Figure S2: Integrin α6β4 is expressed on the surface of HUVECs; Table S1: Antibodies; Table S2: RNAi; Table S3: QPCR Primers; Table S4: Summary of Gene Expression Analysis.
