# Supplementary material for: Contribution of Endothelial Laminin-Binding Integrins to Cellular Processes Associated with Angiogenesis
Source: Cells. 2022 Feb 26;11(5):816. doi: 10.3390/cells11050816 (PMC8909174; doi:10.3390/cells11050816)
Supplement: Supplementary file 1 [file cells-11-00816-s001.zip › cells-1585325-supplementary.pdf]

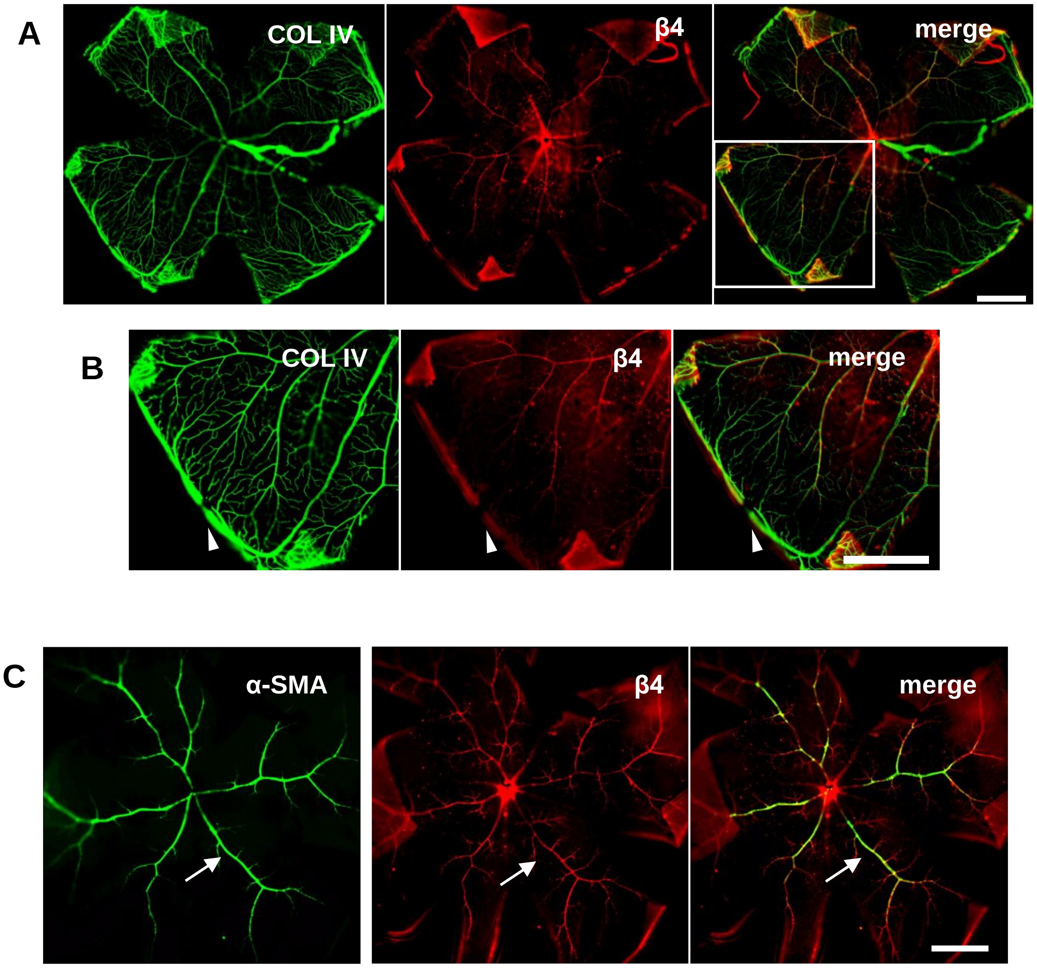

Supplementary Figure S1. Integrin α6β4 is primarily expressed in the arterial vessels of adult mouse retina. (**A**–**B**) Integrin α6β4 is expressed in a subset of vessels of adult mouse retina. (**A**) Immunofluorescence staining of whole mount mouse retina for Collagen IV and β4-integrin subunit. (**B**) Enlarged image of one petal of retina showing restricted β4 expression.
(**C**) Immunofluorescence staining of whole mount mouse retina for α-smooth muscle actin (α-SMA) and β4-integrin subunit. Arrows show examples of α-SMA / β4 co-localization in the arterial branch. of Scale = 1 mm.

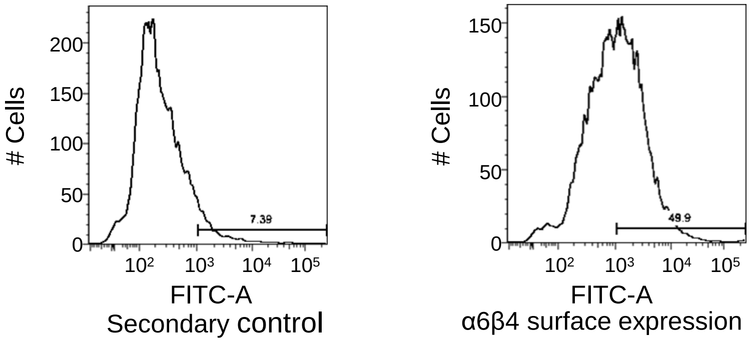

Supplementary Figure S2. Integrin $\alpha 6\beta 4$ is expressed on the surface of human umbilical endothelial cells (HUVECs). HUVECS were immunostained with antibodies to the integrin β4 subunit and analyzed by flow cytometry.

Table S1. Antibodies.

Protein	Antibody/Application*	Manufacturer
CD31 [JC70A, M0823]	Mouse anti-human &mouse/IFM	DAKO
CD31[M-20]	Rabbit anti-mouse /IFM	Santa Cruz
FITC-conjugated -SMA [1A4, A#5228]	Mouse anti-human/mouse/IFM	Sigma
Integrin b4 subunit [346-11A, 553745]	Rat anti-mouse/IFM	BD Biosciences
Integrin b4 subunit [439-9B, 55719]	Rat anti-human/IFM	BD Biosciences
Integrin b4 subunit [# sc-55514]	Mouse anti-human/WB	Santa Cruz
Laminin 4 chain [3H2, ab205568]	Mouse anti-human	Abcam
Laminin 5 chain [4B12, ab77175]	Mouse anti-human	Abcam
Integrin 3 subunit	Mouse anti-human/WB**	Gift from Dr. CM DiPersio
GAPDH [GA1R] [MA5-15738]	Mouse anti-human&mouse/WB	ThermoFisher Scientific
TRITC – UEA Lectin (L4889)	NA/IFM	Sigma
Anti-mouse IgG Alexa Fluor 488 [A21202]	Donkey anti-mouse IgG/IFM	ThermoFisher Scientific
Anti-mouse IgG Alexa Fluor 568 [A10037]	Donkey anti-mouse IgG/IFM	ThermoFisher Scientific
Anti-rabbit IgG Alexa Fluor 488 [A11034]	Goat anti-rabbit IgG/IFM	ThermoFisher Scientific
Anti-Rat IgG Alexa Fluor 647 [A-21247]	Goat anti-rat IgG/IFM	ThermoFisher Scientific
Anti-mouse HRP [ab97046]	Rabbit anti-mouse IgG/WB	Abcam

*IFM = Immunofluorescence microscopy; WB = western blotting.

Table S2. RNAi.

Gene	Manufacturer	Targeting Sequence
Negative Control #1 (siRNA)	Sigma	Sigma SIC001
ITGA3 #1 (siRNA)	Sigma	5′-GUUUGAAGGCUUGGGCAAA-3′
ITGA3 #2 (siRNA)	Sigma	5′-GUGUACAUCUAUCACAGUA-3′
ITGA3 #3 (siRNA)	Sigma	5′-GAGAUCACCGUCCAUGGCA-3
ITGB4 #1 (siRNA)	Sigma	5′-GUCACAUGGUGGGCUUUAA-3′
ITGB4 #2 (siRNA)	Sigma	5′-GCGACUACACUAUUGGAUU-3′
ITGB4 #3 (siRNA)	Sigma	5′-GCACGUGUGAGGAAUGCAA-3′
Non-targeting NT (shRNA)	Dharmacon	5′-TGGTTTACATGTTGTGTGA-3′
ITGA3 (shRNA)	Dharmacon	5′-GTGTTCGTCACGTTGATGC-3′

Table S3. QPCR Primers.

Gene	Forward Sequence (5′-3′)	Reverse Sequence (5′-3′)
LAMA4*	GAAATTGCATTTGAAGTCCG	ACCTGTCCATTTTTCATGTG
LAMA5*	GAAGTGAAAACTCAAGCGGG	CATCGACATACAGCCAGACTC
ITGA3*	GGGGGAATACAAAAAGCCCA	ACCCGTTATTATGTCACGCCA
ITGB4*	AGACCGATCCACTTTGCCAG	AAGTTCAGCCGGCTCTATCG
CXCR4#	AAATCTTCCTGCCCACCATC	GTACTTGTCCGTCATGCTTCT
ANGPT2#	CAAAATAAGCAGCATCAGCCA	AGTACATTCCGTTCAAGTTGGA
DLL4#	CCAACTGCCCTTCAATTTCAC	GGATGGCGATCTTGCTGAT
JAG1#	ACTATGCCTGTGACCAGAATG	CTTAGGACTGCAGCCTTGTC
JAG2#	GTCATCCCCTTCCAGTTCG	GTGGTATCGTTGTCCCAGTC
ID1#	GCTGTTACTCACGCCTCAAG	CCTGATGTAGTCGATGACGTG
ID2#	ATCAGCATCCTGTCCTTGC	CTTAAAAGATTCCGTGAATTTGTTGT
PDGFB#	GAGTTGGACCTGAACATGAC	AGATCTCGAACACCTCGGT
KDR#	TGGAATTGACAAGACAGCAAC	GAGGATCTTGAGTTCAGACATGAG
MMP14#	TTCGCCGACTAAGCAGAAG	CTTGAATTCCTAGACCGCTGT
NRP1#	GCAGTATTCCCCAAACCACT	ATGATCTGGGTCATCTTCTC
ACTB*	GACGACATGGAGAAAATCTG	ATGATCTGGGTCATCTTCTC

*Primers were purchased from Sigma. #Primers were purchased from Integrated DNA Technologies.

Table S4. Summary of Gene Expression Analysis.

Gene	α6 siRNA ‡	β4 siRNA#1	β4 siRNA#2	β4 siRNA#3	α3 siRNA #1	α3 siRNA #2	α3 siRNA #3
LAMA5	P< 0.01 ↓	P< 0.05 ↓	P< 0.05 ↓	NS	NS	NS	NS
LAMA4	NS*	NS	NS	NS	NS	NS	NS
CXCR4	P< 0.01 ↓	NS	NS	NS	NS	NS	NS
ANGPT2	P< 0.01 ↓	P< 0.01 ↓	P< 0.01 ↓	P< 0.05 ↓	NS	NS	NS
DLL4	NS*	NS	P< 0.05 ↓	NS	NS	NS	NS
JAG1	NS	NS	NS	NS	P< 0.01 ↓	NS	P< 0.01 ↓
JAG2	NS	NS	NS	NS	P< 0.05 ↓	P< 0.01 ↓	NS
KDR	NS	NS	NS	NS	P< 0.01 ↓	P< 0.05 ↓	NS
MMP14	ND#	ND	ND	ND	NS	P< 0.05 ↓	NS
NRP1	NS	P< 0.05 ↓	P< 0.001 ↓	P< 0.05 ↓	P< 0.001 ↓	P< 0.001 ↓	P< 0.001 ↓
ID1	P< 0.01 ↑	NS	NS	NS	P< 0.001 ↓	P< 0.05 ↓	P< 0.001 ↓
ID2	P< 0.05 ↓	NS	NS	NS	P< 0.05 ↓	NS	NS
PDGFB	NS	P< 0.001 ↓	P< 0.001 ↓	NS	P< 0.01 ↓	P< 0.05 ↓	NS

*NS = not significant; #ND = not determined; ↓ = RNA expression downregulated; ↑ = RNA expression upregulated; ‡Data is from Xu H,

Pumiglia K, LaFlamme SE. Laminin-511 and alpha6 integrins regulate the expression of CXCR4 to promote endothelial morphogenesis.

J Cell Sci. 2020;133(11).
